# Environmental footprint of gastrointestinal endoscopy services: a systematic review

**DOI:** 10.1055/a-2739-4080

**Published:** 2025-12-19

**Authors:** Britta Vegting, Demi Gerritsen, Ceyda B. Izci, Nicole G.M. Hunfeld, Erik M. van Raaij, Wilco van den Heuvel, Pieter J.F. de Jonge, Peter D. Siersema

**Affiliations:** 1 6993 Department of Gastroenterology and Hepatology, Erasmus MC University Medical Center Rotterdam, Rotterdam, Netherlands; 2 6993 Department of Intensive Care, Erasmus MC University Medical Center Rotterdam, Rotterdam, Netherlands; 3 6993 Department of Hospital Pharmacy, Erasmus MC University Medical Center Rotterdam, Rotterdam, Netherlands; 4 Erasmus School of Health Policy and Management, Erasmus University Rotterdam, Rotterdam, Netherlands; 5 Econometrics Institute, Erasmus School of Economics, Erasmus University Rotterdam, Rotterdam, Netherlands

## Abstract

**Background:**

Gastrointestinal (GI) endoscopy is a significant contributor to health care-related climate change due to high procedure volumes, intensive decontamination processes, and reliance on single-use products. This systematic review aimed to synthesize the current evidence on the environmental impact of GI endoscopy.

**Methods:**

MEDLINE, Embase, and Web of Science were systematically searched up to May 2025 for studies assessing the environmental impact of GI endoscopy. Two reviewers independently performed study selection, data extraction, and quality assessment. The PRISMA guidelines were followed.

**Results:**

28 studies were included. Most studies assessed carbon emissions; only four studies (14%) examined environmental impacts beyond greenhouse gas emissions. The largest contributors to emissions were patient travel, energy use, and procedure-related products, whereas waste had limited impact. Overall, scope 3 emissions accounted for the majority of total emissions, though reporting across different emission scopes was inconsistent. In line with heterogeneity in methodology, per-procedure emissions ranged from 5.4 to 73.2 kg carbon dioxide equivalent. Overall, 21 studies (75%) were judged to have a high risk of bias.

**Conclusion:**

Current evidence on the environmental impact of GI endoscopy services is fragmented, methodologically inconsistent, and often limited in coverage. Emissions were dominated by patient travel, energy use, and procedure-related products. Broader and more standardized environmental assessments are needed to guide the transition to low-carbon, sustainable GI endoscopy.

## Abstract (de)

**Graphical Abstract:**

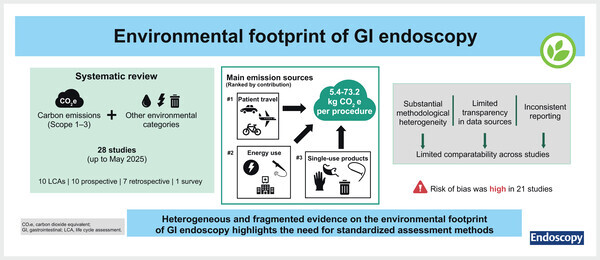

## In Brief

The environmental impact of gastrointestinal endoscopy is summarized in this systematic review of 28 studies. Methodological heterogeneity was very high across studies, preventing firm conclusions being drawn. However, emissions were dominated by three main causes – patient travel, energy use, and procedure-related products – which should be the targets of future intervention.

## Introduction

The health care industry is known to have a substantial impact on the environment through its use of resources (such as minerals, metals, fossil fuels, and fresh water), waste generation, and pollution of air, soil, and water 1 . More specifically, the health care sector is responsible for approximately 5% of global greenhouse gas (GHG) emissions, contributing significantly to climate change with serious threats to ecosystems and human health 1 .

Gastrointestinal (GI) endoscopy contributes considerably to health care-related climate change, primarily due to its resource-intensive decontamination procedures, substantial waste production, high volume of procedures, and reliance on single-use, nonrecyclable products 2 3 . However, the environmental impact, or “environmental footprint,” of GI endoscopy remains incompletely quantified.

Environmental impact of health care services is commonly assessed using carbon footprinting and life cycle assessment (LCA). Carbon footprinting focuses on global warming potential (GWP), quantifying GHG emissions in carbon dioxide equivalents (CO₂e) 4 5 . Emissions are typically categorized by the GHG Protocol into Scope 1 (direct emissions from facility-controlled sources), Scope 2 (indirect emissions from purchased energy), and Scope 3 (all other indirect emissions, including supply chains, travel, and waste) 6 . Scope 3 emissions cover over 70% of health care-related GHG emissions 7 . An LCA offers a more comprehensive approach, evaluating environmental impact across defined stages of a product’s or process’s life cycle, which may extend from raw material extraction to its disposal (“cradle-to-grave”) in accordance with International Organization for Standardization (ISO) 14040 and ISO 14044 standards 8 . It involves defining a functional unit, setting system boundaries, and compiling an inventory of inputs and outputs using process-based or financial activity data. Environmental impacts are then quantified and categorized across multiple dimensions such as GHG emissions, ecotoxicity, and resource depletion 9 . Results are analyzed in terms of completeness, consistency, sensitivity, and uncertainty. Both LCA and carbon footprinting can identify environmental hotspots and support environmental performance over time.

Recognizing the need for sustainable practices in GI endoscopy, the European Society of Gastrointestinal Endoscopy (ESGE) and the European Society of Gastroenterology Nurses and Associates (ESGENA) issued a position statement in 2022 10 . This statement calls for greater awareness of the environmental footprint of GI endoscopy and provides guidance on reducing its environmental impact. It also emphasizes the necessity of high-quality research to quantify the environmental impact of GI endoscopy and to develop actionable strategies for mitigating its environmental footprint.

Published reviews on the environmental sustainability of GI endoscopy predominantly focus on carbon emissions, often without comprehensively addressing the broader environmental impact 11 12 13 14 15 . Moreover, these reviews in general lack a systematic methodology and insufficiently appraise the quality of the studies included.

The present systematic review aimed to provide a comprehensive overview of the existing literature on the environmental impact of GI endoscopy services. Special attention has been given to the carbon footprint of GI endoscopy, with emissions categorized across emission scopes 1, 2, and 3. By synthesizing the available evidence, this review identifies key contributors to the environmental footprint of GI endoscopy and key knowledge gaps to inform future research and sustainability efforts in this field.

## Methods

### Eligibility criteria and outcomes

This systematic review was conducted in accordance with the Preferred Reporting Items for Systematic Reviews and Meta-analysis (PRISMA) guidelines 16 . The study protocol was registered in the International Prospective Register of Systematic Reviews (PROSPERO, National Institute for Health and Care Research, Centre for Reviews and Dissemination, University of York, York, UK), identification number CRD420250599809. A glossary of terminology used in this systematic review is provided in Table 1 .

**Table:** **Table 1** Glossary of terminology used in this systematic review.

Term	Definition/description
Carbon dioxide equivalent (CO _2_ e) ^1^	Standardized metric to quantify emissions of various greenhouse gases (GHGs) based on their global warming potential relative to carbon dioxide (CO _2_ )
Carbon footprint ^1^	Total set of GHG emissions generated directly and indirectly by an individual, event, organization, or product
Endoscopy device	Products typically used during endoscopy procedures (e.g. biopsy forceps, polypectomy snare, hemostatic clips)
Environmental footprint	Method that quantifies the amount of natural resources consumed by an individual, event, organization, or product; can be broken down into multiple impact categories, such as resource depletion, land use, or toxicity 17
Fossil fuel ^1^	Fuel derived from fossilized hydrocarbon deposits, primarily composed of carbon. Examples include coal, petroleum, and natural gas
Functional unit ^1^	The measure of a product or system determined by the performance it delivers in its intended use (i.e. item or process that is being measured)
Global warming potential (GWP) ^1^	Measure developed to quantify the warming effects of various gases relative to CO _2_ emissions. A GWP >1 indicates that a particular gas has a greater warming effect on Earth compared with CO _2_ during that specific timeframe (usually 100 years)
Greenhouse gases (GHGs) ^1^	Atmospheric elements that absorb and release radiation at particular wavelengths within the range of terrestrial radiation emitted by the Earth’s surface, the atmosphere, and clouds. This characteristic leads to the greenhouse effect. Key GHGs include water vapor, CO _2_ , nitrous oxide, methane, and ozone
ISO 14040/14044 standards ^1^	International Organization for Standardization (ISO) refers to a worldwide federation of national standards bodies. In this particular case, ISO 14040/14044 refers to international standards that cover life cycle assessment (LCA) studies
Landfill waste ^1^	Landfill waste refers to solid waste materials such as nonrecyclable items (plastic bags, food waste, paper products, and other household waste) that are disposed of in specially designed areas called landfills; also, in the present context, nonrecyclable endoscopy supplies not contaminated with body fluids
Life cycle assessment (LCA) ^1^	Methodology that systematically evaluates the environmental factors and potential consequences of product systems through a “cradle-to-grave” or “cradle-to-gate” analysis, spanning from obtaining raw materials to their ultimate disposal, according to specified objectives and boundaries
LCA goal and scope ^1^	*First phase of an LCA* : Includes the specifying principles (functional unit and system boundaries), requirements, and guidelines to assess the environmental impact of products, processes, and organizations
Life cycle inventory (LCI) analysis phase ^1^	*Second phase of an LCA* : Compilation and quantification of data inputs and outputs for a product or service throughout its life cycle, necessary to meet the goals of the defined study
Life cycle impact assessment (LCIA) phase ^1^	*Third phase of an LCA* : Evaluation of the scale and importance of potential environmental impacts associated with a product system over its entire life cycle. In this phase, LCI results are assigned to impact categories, with specific emissions and resource usages linked to broader environmental and human health impacts. These results provide insights into the environmental concerns linked with both the inputs and outputs of the product system
Life cycle interpretation ^1^	*Final phase of an LCA* : Summary and discussion of LCI and/or LCIA results in relation to the defined goal and scope, in order to reach conclusions and recommendations
Life cycle model	Model to determine what life cycle stages (raw material extraction, also called “cradle,” manufacturing and processing, transportation, usage and retail, waste disposal, also called the “grave”) are covered in an LCA, structuring the process of data collection and analysis 8
Cradle-to-gate	Model for assessment of the manufacturing process of a product, covering the product life cycle from raw material extraction (“cradle”) up to the product’s departure from the manufacturing facility (“gate”) 8
Cradle-to-grave	Model for comprehensive assessment of the life cycle of a product, from raw material extraction (“cradle”) up to its disposal (“grave”) 8
Material	A physical substance that objects (products) can be made from
Product	An article or substance that is manufactured or refined for sale. A product is made of one or more materials
Single-use product	Products that are used once, or for a short period of time before being discarded or recycled
Reusable product	Products that can be used multiple times for their intended purpose or a different purpose, rather than being discarded after a single use
Regulated medical waste ^1^	Nonrecyclable items saturated with body fluids or containing infectious agents
Scopes 1, 2, and 3 ^1^	*Scope 1:* Direct emissions (e.g. fuel combustion for boilers or vehicles, CO _2_ insufflation) *Scope 2:* Indirect emissions associated with the purchase of electricity (e.g. for heating, ventilation, or cooling) *Scope 3:* Indirect emissions generated within the supply chain of endoscopic supplies (manufacturing, transportation, and disposal)
System boundary ^1^	A defined set of criteria for selecting the unit processes that form a product system
^1^ Definitions adopted from Cunha Neves et al. (2025) 18 .	

We included peer-reviewed studies assessing the environmental impact of GI endoscopy, with no restrictions on department size or geographical location. Included studies addressed at least one of the 16 environmental impact categories, as defined by the European Commission 9 , or addressed waste, patient or staff travel, or energy consumption. Only studies presenting original data, published in English, and with full-text access were included. Studies comparing endoscopy services with other care pathways were not included.

The primary outcome was the environmental footprint of GI endoscopy departments, categorized according to the European Commission’s environmental footprint impact categories. Secondary outcomes included the comparison of GHG emissions across three emission scopes (Scopes 1, 2, and 3), with a focus on identifying key environmental hotspots, and identifying opportunities for future environmental impact studies in the field of GI endoscopy.

### Search strategy

A comprehensive literature search was conducted by a professional librarian across MEDLINE, Embase (OVIDSP), and Web of Science databases through November 12, 2024, with an updated search through May 25, 2025. Custom search queries were developed for each database. The following search terms were used: endoscopy, digestive endoscopy or digestive tract endoscopy, different types of GI endoscopy procedures, combined with environment, climate change, global warming, GHG, carbon emissions, carbon footprint, pollution, sustainability, fossil fuels, and specific environmental footprint impact categories such as particulate matter, ionizing radiation, ocean acidification, eutrophication, ozone depletion, land use, soil quality, ecotoxicity, water use, resource use, or waste disposal. Reference lists of included studies and relevant reviews were screened for additional eligible studies. A detailed list of the search strategy is shown in **Table 1s** .

### Study selection

**Fig. 1:**
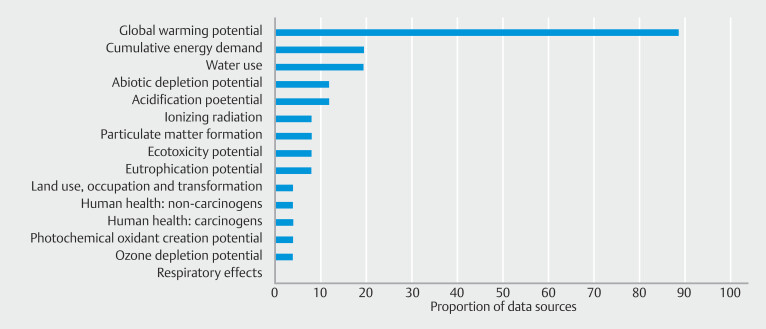
Environmental impact categories assessed in included studies. Presented as percentages of total included studies (N = 28).

Duplicate records were removed using Endnote (Clarivate Analytics, Philadelphia, Pennsylvania, USA) and screened using Covidence software (Covidence systematic review software; Veritas Health Innovation, Melbourne, Victoria, Australia). Two reviewers (BV, DG) independently screened titles and abstracts. Full-text articles were then assessed for inclusion, with disagreements resolved by consensus. Reasons for exclusion were documented and are summarized in **Fig. 1s** .

### Data extraction

Data extraction was performed in duplicate by two reviewers (BV, DG), including extraction of study details, coverage, environmental assessment methods, system boundaries, and environmental outcomes. Environmental impact was quantified as results from one or more environmental impact categories. For example: the impact category GWP, measured in GHG emissions, was recorded in kilograms (kg) of CO _2_ e. GHG emissions were further categorized by GHG emission scope. Results beyond GHG emission scope such as energy consumption (kWh) and waste generation (kg) were reported separately. Results from studies reporting on sustainability interventions with two or more data points were reported as a range. Due to methodological heterogeneity, a meta-analysis was not feasible.

### Quality assessment

Risk of bias was assessed using the Center for Environmental Evidence Critical Appraisal Tool (CEECAT), version 0.3 19 . The seven CEECAT criteria were prespecified for endoscopy sustainability studies and independently rated by two reviewers (BV, DG), with discrepancies resolved by consensus. Studies were classified as low, medium, or high risk of bias, with overall risk determined by the highest score. To address methodological variability, the ESGE recently published a position statement outlining minimum criteria for environmental impact assessments in GI endoscopy, including a checklist to guide study design, reporting, and interpretation (E-SPARE) 18 . This checklist was used by two reviewers (BV, CBI) to assess these criteria for all included studies. Additionally, for studies reporting LCAs, a pro forma quality assessment scoring system adopted from Drew et al. (2021) was used, based on Weidema’s guidelines for critical review of LCAs and operationalized by Kouwenberg et al. 20 21 22 . This scoring system consists of 16 appraisal criteria covering the four phases of LCA and addresses a range of quality indicators, including internal and external validity, transparency, consistency, and bias. A maximum of 35 points could be allocated. Points were assigned for each study by two reviewers (BV, CBI), and a score out of 35 was calculated to provide an indication of overall study quality. All discrepancies were resolved by consensus.

## Results

### Study characteristics

A total of 2939 references were identified through database searches ( **Fig. 1s** ) and one through citation screening. After removal of 1172 duplicates, 1768 records underwent title and abstract screening. A total of 132 abstracts appeared relevant, and the full papers of these abstracts were assessed. After application of the inclusion and exclusion criteria, a total of 107 articles were excluded, including 17 studies focusing on direct radiation exposure and 6 on room air quality.

A total of 28 studies were finally included 23 24 25 26 27 28 29 30 31 32 33 34 35 36 37 38 39 40 41 42 43 44 45 46 47 48 49 50 . These articles were published between 2008 and 2025 (79% in 2023 or later) and originated from Europe (19 studies), the USA (5 studies), and Australasia (4 studies). The studies were primarily conducted in tertiary centers. One study was conducted during the COVID-19 pandemic 23 . Full study characteristics and results are summarized in Table 2 .

**Table:** **Table 2** Study characteristics, methods, and outcomes.

Study characteristics			Study methods			Outcomes	
**Author (year) [ref] Country**	**Assessment period and number of procedures assessed**	**Assessment type**	**Setting**	**System boundaries**	**Environmental impact categories assessed**	** Reported GHG emissions (kg CO _2_ e) **	**Other reported measures**
Cunha Neves et al. (2023) 23 Portugal	October 2021 – March 2022 Pre-intervention (T0): 185 endoscopies 1 month after intervention (T1): 178 4 months after intervention (T2): 172	Sustainability intervention study	Waste generated by GI endoscopy during 4 weeks	Included: landfill waste, regulated medical waste (RMW), recycled plastic, recycled paper Excluded: sharps waste, pre- and post-interventional waste, waste due to endoscope reprocessing	GHG emissions Waste generation (kg)	RMW: T0: 362.1 (82.5%) T1: 212.1 (70.7%) T2: 204(70.1%) Total carbon footprint: T0: 438.7 T1: 299.9 T2: 286.6	Landfill waste: T0: 76.6 kg (38.8%) T1: 87.8 kg (51.2%) T2: 82.6 kg (50.9%) RMW: T0: 120.7 kg (61.2%) T1: 70.7 kg (41.2%) T2: 68 kg (41.9%) Recycled paper: T0: 0 kg (0%) T1: 4.7 kg (2.8%) T2: 3.8 kg (2.3%) Recycled plastic: T0: 0 kg (0%) T1: 8.2 kg (4.8%) T2: 8 kg (4.9%) Total waste: T0: 197.3 kg T1: 171.4 kg T2: 162.4 kg
De Jong et al. (2023) 24 Netherlands	February 2020; 15 procedures + February 2021: 21 procedures	Sustainability intervention study	Waste generated per endoscopy procedure	GI endoscopy unit with 10 000 procedures per year	GHG emissions Waste generation (kg)	Baseline measurement (T0): 4.69 per procedure After recycling (T1): 4.55 per procedure	T0: Total: 0.97 kg, 85% residual waste, 9.6% recyclable plastic waste T1: 0.89 kg, 8.9% recyclable plastic waste
Desai et al. (2024) 25 USA	May–June 2022; 450 EGDs/ colonoscopies in 400 patients	Prospective study	Waste generation and energy use for 100 procedures	Included: Total waste (landfill, biohazard, potentially recyclable, sharps) of all devices, PPE, packaging, and tubing Liquid waste generated from endoscope reprocessing Energy use of endoscopy unit and endoscopy tower, electrocautery machine, monitors	GHG emissions Water use Waste generation (kg) Energy consumption (kWh)	Emissions for 100 procedures: 1501 Landfill waste: 766.5 Energy consumption: 734.58	For 100 procedures: Waste: 303 kg Direct landfill waste: 219 kg Biohazard: 72.8 kg Sharps: 11.1 kg Recyclable items: 61 kg Endoscope reprocessing: 5243 L Energy consumption: 1980 kWh
Elli et al. (2024) 26 Italy	Unknown	Retrospective study	One upper or lower GI endoscopy procedure	Included: Energy use (including energy required to operate endoscopes, climate, lighting of the endoscopic room, use of computers) Endoscope reprocessing Use of PPE, single-use devices, and products, vascular access Paper to print report and pictures Histology processing Excluded: Energy consumption during manufacture and transportation of materials	GHG emissions, energy consumption (kWh)	EGD: 5.43 Colonoscopy: 6.71	Energy consumption: EGD: 5.5 kWh per procedure Colonoscopy: 11.0 kWh per procedure
Fichtl et al. (2024) 27 Germany	Baseline (T0): 30 days + Power saving phase (T1): 30 days	Sustainability intervention study	Energy use per procedure	Included Energy consumption of endoscopy tower	GHG emissions Energy consumption (kWh)	Center 1: T0: 0.06925 T1: 0.05744 Center 2: T0: 0.15928 T1: 0.14428 Center 3: T0: 0.15357 T2: 0.14212	Mean power consumption per examination: Center 1: T1: 159.56 Wh (±23.91) T1: 132.36 Wh (±20.51) Center 2: T0: 367.01 Wh (±40.65) T1: 332.44 Wh (±62.6) Center 3: T0: 353.84 Wh (±93.66) T2: 327.46 Wh (±74.51)
Gayam (2020) 28 USA	Unknown	Retrospective study	Energy consumption in a single day	Included: Energy consumption of wash machines, endoscopy machines, anesthesia machines, room lighting	GHG emissions Energy consumption (kWh)	Energy use per year: 15 780	Energy use per day: Wash machines: 24.67 kWh Endoscopy machines: 27.00 kWh Anesthesia machine: 12.00 kWh Room lighting: 47.88 kWh Total: 111.55 kWh Energy use per year: 29 003 kWh
Gordon et al. (2021) 29 USA	Unknown	Process-based LCA	Processing of 1 person's biopsy sample	Included: All biopsy materials and supplies used within the laboratory space, associated electricity used, upstream production and downstream treatment or disposal of resources, transportation of staff Excluded: Manufacturing of capital equipment and buildings, nonelectricity energy demand	GHG emissions	1 specimen jar with biopsies: 0.29 3 specimen jars with biopsies: 0.79	N/A
Grau et al. (2025) 30 France	Sep 2019–Feb 2021 P-EMR: 182 ESD: 177 Simulated follow-up period of 18 months	Process-based LCA	P-EMR and ESD procedures	Included: Medical devices, bowel preparation, drugs for anesthesia (only packaging), electricity consumption, patient transport Excluded: Staff travel, impact of outpatient clinics, overnight stay in hospital, meals, endoscopes	GHG emissions Waste generation (kg)	P-EMR: 63.5 (equipment 10.5, patient transport 32.7, electricity 8.0, anesthesia 12.3) 31.3 excluding transport ESD: 73.2 (equipment 13.3, transportation 33.4, electricity 12.5, anesthesia 12.9) 39.3 without transportation Follow-up colonoscopy at local center: 16.5 Follow-up at expert center: 43	Waste per procedure: P-EMR: 1.7 kg ESD: 2.3 kg Waste for one standard simulated follow-up colonoscopy: 0.6 kg
Henniger et al. (2023) 31 Germany	1 February 2022–1 May 2022 and 1 February 2023–1 May 2023 (intervention period); 1738 + 1666 endoscopies	Sustainability intervention study	Waste generated per day	Included: Consumables (transportation, production, waste burning), waste, energy-related emissions	GHG emissions Waste generation (kg)	Control: 8010 Intervention: 7090	Total waste: Control: 70.84 kg/day Intervention: 69.88 kg/day
Henniger et al. (2023) 32 Germany	1 January 2022–31 December 2022 Middle-sized GI endoscopy unit (8000–8500 procedures)	Retrospective study	All procedures in a GI endoscopy unit for 1 year	Included: Electrical power and gas used for heating, waste treatment, endoscopic devices, and protective materials (manufacturing, packaging, transportation: cradle to gate) Excluded: Staff travel needs, capital goods	GHG emissions Energy consumption (kWh)	Total emissions: 62 720 per year Scope 1: Consumption of natural gas: 35 910 Scope 3: 26 810 Materials 14 150 Extraction, processing and transport of natural gas and electricity: 8470 Packaging: 890 Transportation: 2750 Handling waste: 550	Scope 2 (Electricity): 46 622 kWh (from renewable sources, so CO _2_ e = 0 kg)
Jalayeri Nia et al. (2024) 33 UK	December 2022–September 2023 25 patients	Prospective study	P1: CRC screening via conventional colonoscopy P2: Clinical CCE P3: Home-delivered CCE	P1: Patient travel, energy usage, waste disposal, polyp removal, IM morphine used as proxy for sedation and analgesia medicines P2: Patient travel P3: Courier service delivering and collecting the smartbox, staff travel Excluded: Colonoscopy capsules, 5G hardware and smartbox manufacture, bowel preparation, staff travel	GHG emissions	P1: Base case (BC): Travel: 6.62 Procedure: 5.46 Pharma: 0.02 Total: 12.10 Optimized case (OC): Travel: 2.52 Procedure: 3.06 Pharma: 0.02 Total: 5.60 P2: BC: Travel: 17.09 Procedure: 3.87 Pharma: 0.01 Total: 20.98 OC: Travel: 7.99 Procedure: 1.56 Pharma: 0.01 Total: 9.57 P3: BC: Travel: 12.67 Procedure: 3.87 Pharma: 0.01 Total: 16.56 OC: Travel: 1.36 Procedure: 1.56 Pharma: 0.01 Total: 2.94	N/A
Jung et al. (2025) 34 South Korea	5-day audit in October 2023 3922 endoscopies in 7 hospitals	Prospective study	Waste of GI endoscopy procedures in South Korea	Excluded: Specific therapeutic interventions, such as endoscopic resection and stent insertion	Waste generation (kg)	N/A	Total waste: 4558 kg Mean weight per procedure: 1.34 kg Disposable weight per EGD: 0.24 kg (0.05–0.35 kg) Disposable waste per colonoscopy: 0.43 kg (0.12–0.61 kg)
Klose et al. (2024) 35 Germany	January–June 2023 300 procedures in 260 patients	Survey	One outpatient endoscopy procedure	Included: Travel for pre-endoscopic consultation and the endoscopic procedure	GHG emissions	Patients: 10.7 Staff: 0.8	N/A
Kojima et al. (2008) 36 Japan	November 2004 (baseline) Panendoscopies: 45 Colonoscopies: 19 December 2004-November 2005 (intervention) Panendoscopies: 220 Colonoscopies: 87	Sustainability intervention study	N/A	Included waste categories: Sharp infectious waste, needle, infectious waste, noninfectious waste, noninfectious plastic waste	Waste generation (kg)	N/A	Before HACCP implementation: Sharp infectious waste: 6.6 kg (7.1%) Infectious waste: 86.6 kg (92.9%) Total: 93.2 kg After HACCP implementation: Sharp infectious waste: 6.4 kg (6.8%) Needle: 0.2kg (0.2%) Infectious waste: 64.2 kg (68.9%) Noninfectious waste: 17.7 kg (19.0%) Noninfectious plastic waste: 4.6 kg (4.9%) Total: 93.2 kg
Lacroute et al. (2023) 37 France	January 2021–December 2021 8524 procedures for 6070 patients	Retrospective study	Ambulatory endoscopy center	Included: Energy use, medical gases, medical and nonmedical equipment, consumables including, food products, laundry services and cleaning, patient and staff travel, waste Excluded: Manufacturing of products not in database, transportation of products from outside Europe	GHG emissions Waste generation (kg) Energy consumption (kWh)	Total emissions: 241 400 (±56 000) Per procedure: 28.4 Travel: 110 014 (45%) Medical and nonmedical equipment: 77 556 (32%) Energy: 28 937 (12%) Electricity: 3000 Consumables: 17 339 (7%) Waste: 6639 (3%) Freight: 619 (0.4%) Medical gases: 11 (0.005%)	Electricity: 57 840 kWh Waste: 1.5 kg per procedure
Lämmer et al. (2025) 38 Netherlands	July 12–27, 2023 13 colonoscopies	Process-based LCA	Diagnostic colonoscopy procedures	Included: Extraction of raw materials, production, transport, use phase, waste processing, reprocessing, energy, and water use Excluded: Hospital infrastructure and medical gas infrastructure	GHG emissions Raw material extraction Water use Human carcinogenic toxicity, human health	56.4 per colonoscopy Excluding transport: 14.2 per colonoscopy	Human health damage: DALYs per colonoscopy: 11.3·10 ^-5^ Water consumed: 137 L Transportation of patients/staff: 76.5% of total Disposables: 13.5%
Le et al. (2022) 39 USA	Unknown	Process-based LCA	One ERCP using one of three duodenoscopes: Conventional RD RD with disposable endocaps SD	Included: Manufacturing, transportation, and packaging Disposal, cleaning, infection treatment Electricity during use Excluded: Recycling of SDs	GHG emissions Acidification Eutrophication Resource depletion Ionizing radiation Ozone depletion Water use Ecotoxicity Land use Human health	Performing an ERCP with an SD: 36.3–71.5 (91%–96% manufacturing, 3%–5% disposal) RD: 1.53 (electricity use 62%, cleaning and disinfection 26%) RD with disposable endcap: 1.54	Human health (DALY): DALY for RD: 2.31E-04 RD with disposable endcap: 1.15E-04 Other outcomes (end point): RD: Human health DALY (DALY): 1.31E-05 Ecosystem quality species per year (EQSy): 6.22E-08 Resource consumption USD2013 (RCusd): 8.50E-02 RD with disposable endcaps: DALY: 1.29E-05 EQSy: 6.12E-08 RCusd: 8.53E-02 SD (lower bound): DALY: 1.70E-04 EQSy: 2.58E-07 RCusd: 2.24E+00 SD (upper bound): DALY: 3.42E-04 EQSy: 4.67E-07 RCusd: 4.28E+00
López-Muñoz et al. (2025) 40 Spain	RD: 1 600 procedures SD: 1600 procedures Combination of 1405 uses of an RD plus 195 procedures using SDs	Process-based LCA	One ERCP procedure	Excluded: Electricity consumption during ERCP, medical and nonmedical equipment, other consumables, general waste, travel	GHG emissions Acidification Ionizing radiation Water use Resource depletion	Emissions per one endoscopy: RD: 0.1 SD-A: 7.9 SD-B: 6.6 Emissions for one endoscopy when endoscope is used 1600×: RD: 152 SD-A: 12 640 SD-B: 10 512 Reusable + single use A (1405× RD + 195× SDs): 1677 Reusable and single use B (1405× RD + 195× SDs): 1417	RD: Acidification (Ac): 0.16 mol H+ eq Water use (WU): 7.17m ^3^ Resource use (RU): 0.00116 kg Sb-eq Ionizing radiation (IR): 0.95 kg 235U-eq SD-A: Ac: 0.02 mol H+ eq WU: 1.31m ^3^ RU: 0.00012 kg Sb-eq IR: 0.15 kg 235U-eq SD-B: Ac: 0.011 mol H+ eq WU: 0.91 m ^3^ RU: 0.00012 kg Sb-eq IR: 0.15 kg 235U-eq
López-Muñoz et al. (2023) 41 Spain	June 2022–July 2022 Devices: 143 Biopsy forceps: 75 Polypectomy snares: 49 Hemostatic clips: 19 to assess the efficacy of a “green mark”	Process-based LCA + 1-week prospective sustainability intervention study	Devices from 4 manufacturers (A, B, C, and D) Biopsy forceps (A, B, and C) Polypectomy snares (A, B, and D) Hemostatic clips (A and B)	Included: Extraction of material and energy resources Manufacturing, transport of production process and disposal Weight and composition of endoscopy devices Excluded: Manufacturing and assembly steps (injection, extrusion, and lamination) were not included (around 15% of total)	GHG emissions	Hemostatic clips: 0.49 (range 0.41–0.57) Snares: 0.41 (range 0.38–0.44) Forceps: 0.41 (range 0.31–0.47) Total: 67.74 After intervention: –18.26 (–27.44%)	N/A
Lotter et al. (2025) 42 Australia	77 342 sterile water bottles	Process-based LCA	Sterile water bottles used for colonoscopy	Included: Sterile water bottle manufacturing, transport, and disposal Excluded: Transport of waste, oil used to produce bottles, transport of bottles in the region	GHG emissions	Total 77 342 bottles: Landfill 15 247 Recycling 23 035 Incineration 31 330 Per bottle: Landfill 0.197 Recycling 0.298 Incineration 0.405	N/A
Martin-Cabazuelo et al. (2024) 43 Spain		Process-based LCA	Snares (S1–3) Hemostatic clips (H1, H2) Biopsy forceps (F1–3)	Included: Production, assembly, transportation, waste management Excluded: Sterilization, user manuals	GHG emissions	S1 0.72, S3 0.52 F1 0.69, F3 0.48 H1 0.54, H2 0.80	N/A
Namburar et al. (2022) 44 USA	5-day audit in January and February 2020 278 endoscopies for 243 patients	Retrospective study	One endoscopy procedure	Included: Pre- and post-procedure care Excluded: Waste from patient waiting areas, staff break rooms, and sharps waste	Waste generation (kg)	N/A	Total: All: 619 kg Low-volume center (LVC): 73 kg High-volume center (HVC): 546 kg Per endoscopy: All: 2.11 kg LVC: 1.96 kg HVC: 2.27 kg Landfill: All: 1.34 kg (64%) LVC: 1.33 kg (68%) HVC: 1.36 kg (60%) Biohazard: All: 0.59 kg (28%) LVC: 0.64 kg (32%) HVC: 0.54 kg (24%) Recycled: All: 0.18 kg (9%) LVC: 0 kg (0%) HVC: 36 kg (16%) Reprocessing: All: 0.30 kg LVC: N/A HVC 0.33 kg
Pioche et al. (2024) 45 France	November 2022–February 2024 100 patients Three devices: PillCam (PC) CapsoCam (CC) NaviCam (NC)	Process-based LCA; survey	One small-bowel capsule endoscopy procedure	Included: Materials, packaging manufacturing, transport, use, disposal, bowel preparation, patient and staff transport, data storage, capsule retrieval Excluded: Water to flush toilet, capsule journey	GHG emissions	PC: 19.4 CC: 20.6 NC: 19.5 Including consultations: PC: 27.2 CC: 28.5 NC: 27.3 All packaging components recycled: PC: –0.09 CC: –0.13 NC: –0.06	N/A
Pioche et al. (2024) 46 France	April 2023–February 2024	Hybrid LCA	Provision of an endoscope for 1 upper GI endoscopy	Included: Manufacture, distribution, usage, reprocessing and disposal of endoscope Excluded: Pre- and post-care, patient and staff travel, sedation, bite block, lighting and energy, additional devices (e.g. forceps)	GHG emissions Acidification Eutrophication Resource depletion Water use Ecotoxicity	Single-use gastroscope (SG): Total: 10.9 Component production: 5.7 Assembly and sterilization: 1.4 Supply manufacturer: 0.2 Supply distributor: 0.1 Packaging: 1.5 End-of-life treatment: 2.1 Reusable gastroscope (RG): Total: 4.7 Endoscope production and assembly: 0.02 Primary packaging: 0.4 Supply: 0.05 Decontamination: 2.1 Sent for repair: 0.06 Sampling: 0.01 End-of-life treatment: 2.1	SG: Depletion fossil resources (DFR): 130 MJ Freshwater ecotoxicity (FE): 15.9 kg 1,4-DB _e_ Terrestrial acidification (TA): 0.12 kg SO _2_ e Eutrophication (Eu): 0.02 kg PO _4_^3-^ e Water consumption (WC): 6.2 M ^3^ RG: DFR: 60.9 FE: 2.6 TA: 0.02 Eu: 0.005 WC: 9.5
Ribeiro et al. (2024) 47 Portugal	14–18 February 2022, 241 procedures	Prospective study	Waste generated during 1 endoscopy procedure	Included: Mass of waste from pre- and post-procedural areas, endoscopy rooms, as well as the reprocessing area + the amount of water used during the reprocessing of a single endoscope	Water use Waste generation (kg)	N/A	Total waste: 443.2 kg Endoscopy rooms: 310.8 kg (70%) Pre- and post-procedural area: 55.2 kg (13%) Reprocessing: 77.2 kg (17%) Waste per procedure: 1.8 kg, of which 1.4 kg hazardous (group III) Water consumption: 250 mL for precleaning, 30 L for manual cleaning and rinsing (15 L for each), 25 L high-level disinfection Total (241 procedures): 13 315.3 L of water (55.3 L per endoscope)
Rughwani et al. (2025) 48 India	29 May–10 June 2023 3873 procedures in 3244 patients	Prospective study	GI Endoscopy department	Included: Electricity use, water use (reprocessing and laundry), waste, patient travel, medical gas, transport of endoscopes and devices, detergents and disinfectants, laundry Excluded: Manufacturing of consumables, endoscopes, and medical gases	GHG emissions Waste generation (kg) Energy consumption (kWh) Water use	Total emissions: 148 947.32 or 38.45 per procedure Patient travel: 83.09% Electricity consumption 10.42% Medical gas transport and usage 3.63% Water consumption 1.86%	Waste: Total: 1952.50 kg Per procedure: 0.504 kg Electricity: Total: 19 160.4 kWh Per procedure: 4.94 kWh Water use: 67.85 L per procedure
Vaccari et al. (2018) 49 Italy	2013 and 2014 (2 years)	Retrospective study	Hospital waste	Included: Nonhazardous healthcare waste including unsorted municipal waste, organic waste and paper/cardboard	Waste generation (kg)	N/A	Total: 0.50 kg/procedure Hazardous waste: 3.09 kg/day/bed
Zullo et al. (2023) 50 Italy	2000 hypothetical upper endoscopy procedures	Retrospective study	Upper GI biopsy sampling for one patient	Included: Bottles for calibration plus a liquid-draining system, cardboard box for the 3 bottles, washing solution tank, gastric juice suction tube, histology assessment, biopsy forceps, biopsy jar Excluded: Calibration liquids and reagents	GHG emissions	Standard biopsy sampling: 1262 per year EndoFaster: 704 per year	N/A
Please refer to main text for details on references. 1,4 DB _e_ , 1,4-dichlorobenzene equivalent; CCE, colon capsule endoscopy; CO _2_ e, carbon dioxide equivalent; DALY, disability-adjusted life year; EGD, esophagogastroduodenoscopy; ERCP, endoscopic retrograde cholangiopancreatography; ESD, endoscopic submucosal dissection; GHG, greenhouse gas; GI, gastrointestinal; HACCP, hazard analysis and critical control points; IM, intramuscular; kg, kilogram; kWh, kilowatt-hour; L, liter; LCA, life cycle assessment; M, meter; MJ, megajoule; N/A, not applicable; P-EMR: piecemeal endoscopic mucosal resection; PO _4_^3-^ e, phosphate equivalent; PPE, personal protective equipment; RD, reusable duodenoscope; SD, single-use duodenoscope; SO _2_ e, sulfur dioxide equivalent; USD, United States dollars.							

### Study design and methodology

#### Study design

Of the 28 studies, 10 used LCAs 29 30 38 39 40 41 42 43 45 46 , 10 were prospective studies 23 24 25 27 31 33 34 36 47 48 with five of them focusing on sustainability interventions 23 24 27 31 36 , seven were retrospective studies 26 28 32 37 44 49 50 , and one reported a survey 35 . Overall, 23 studies assessed one or more environmental impact categories, with GWP reported in all ( Fig. 1 ) 23 24 25 26 27 28 29 30 31 32 33 35 37 38 39 40 41 42 43 45 46 48 50 . Fresh water use 25 38 39 40 46 47 48 and energy consumption 25 26 27 28 32 37 48 were both assessed in seven studies. Seven studies covered entire departments 32 37 48 or procedures 26 33 38 45 , while 11 focused on specific products, including capsule endoscopy 33 , endoscopy devices 41 43 , and endoscopes 39 40 46 .

#### System boundaries

**Fig. 2:**
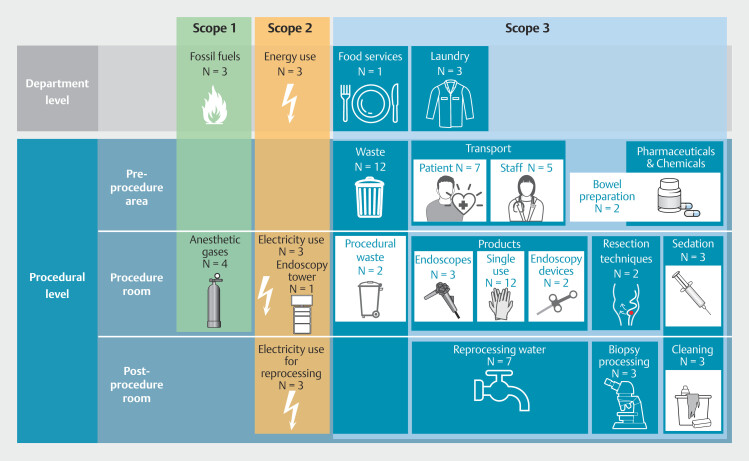
Distribution of studies across Greenhouse Gas Protocol Scopes 1–3 in gastrointestinal endoscopy, mapped by procedural stage and departmental level.

A total of 12 studies adopted a “cradle-to-grave” approach, while two used “cradle-to-gate,” meaning the coverage of the life cycle of products only up to the product’s departure from the manufacturing facility (“gate”). The remaining studies focused on travel, electricity consumption, and/or waste generation. All 10 GHG Protocol components were covered in one or more studies ( Fig. 2 ). Inclusion of Scope 3 emissions was inconsistent, such as patient and staff travel, and manufacture of medical products and pharmaceuticals ( **Table 2s** ).

#### Data sources

Two studies used a hybrid approach, combining financial activity and process data 37 46 , while others used a process-based approach. Emission factors were drawn from a range of data sources, including the GHG Protocol and national databases. Emissions from electricity consumption were based on the energy mix in each country, with one study noting a 32% reduction in CO _2_ emissions when switching to 100% renewable energy 32 . An overview of study methods is presented in **Table 3s** .

### Study results

#### Carbon footprint of entire departments and procedures

Three studies examined the carbon footprint of GI endoscopy departments. Of these, Lacroute et al. reported an annual GHG emission of 241.4 tons CO _2_ e for 2021, or 28.4 kg CO _2_ e per procedure, with the largest contributors being patient and staff travel (45%) and medical and nonmedical products (32%) 37 . Rughwani et al. reported GHG emissions of 3244 patients undergoing 3873 procedures in an ambulatory endoscopy clinic in India, showing a total carbon footprint of 148.9 tons CO _2_ e, or 38.5 kg CO _2_ e per procedure, of which 83% were emissions from patient travel 48 . Henniger et al. reported 62 720 kg CO _2_ e annually in a mid-sized department (8000–8500 procedures per year), equating to 7.8–8.4 kg CO _2_ e per procedure, excluding patient travel and medical and nonmedical products 32 .

Four studies investigated the carbon footprint of a specific endoscopic procedure. Elli et al. reported 5.4 kg CO _2_ e per gastroscopy and 6.7 kg CO _2_ e per colonoscopy, not including travel of patients or staff, or medical products 26 . Lämmer et al. reported 56.4 kg CO _2_ e per colonoscopy, including transport of patients and staff, and 14.2 kg CO _2_ e when excluding transport 38 . Major contributors were transportation of patients and staff (76.5%) and the use of single-use products (13.5%). Another study reported up to 12.1 kg CO _2_ e per colonoscopy, and emphasized the significance of patient travel; colon capsule endoscopy had lower emissions than colonoscopy, resulting in patient travel contributing around 80% of the total emissions 33 . Pioche et al. found even higher numbers for small-bowel capsule endoscopy, with patient travel contributing up to 94.7% of total emissions 45 .

#### Scope 1 and 2 emissions

Three studies evaluated Scope 1 emissions, with heating-related CO _2_ emissions ranging from 2.2 kg CO _2_ e to 4.8 kg CO _2_ e per procedure 32 37 48 . Scope 2 emissions from energy use were assessed in seven studies, with significant variability 25 26 27 28 32 37 48 . Henniger et al. reported zero emissions due to the use of renewable energy while other studies reported electricity-use and related emissions ranging from 0.2–5.5 kWh or 0.1–1.4 kg CO _2_ e per procedure ( **Table 4s** ) 26 27 28 32 48 . One study reported 19.8 kWh or 7.4 kg CO _2_ e per procedure 25 .

#### Scope 3 emissions

**Fig. 3:**
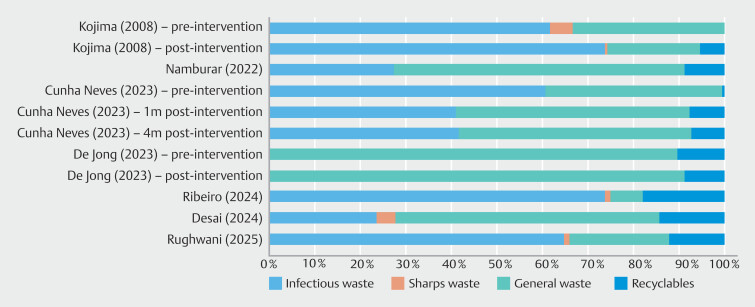
Types of waste and their percentage per waste category, categorized using the World Health Organization standard health care waste categories, excluding pathological, chemical, and pharmaceutical waste, as no study examined these categories.

A total of 24 studies examined some aspects of Scope 3 emissions 23 24 25 29 30 31 33 34 35 36 37 38 39 40 41 42 43 44 45 46 47 48 49 50 . Of these, 10 studies 29 30 33 39 40 41 42 43 46 50 quantified the environmental impact of Scope 3 emissions of medical products, with reusable endoscopes generally having a much lower footprint per procedure than single-use models. For example, Le et al. concluded that single-use duodenoscopes produced 47 times more GHG emissions per procedure than reusable duodenoscopes 39 . Additionally, endoscopy devices such as biopsy forceps and snares generated considerable emissions, with biopsy-related emissions also being notable 41 43 . Resecting colonic adenomas by endoscopic submucosal dissection (ESD) generated almost double the amount of GHG compared with piecemeal endoscopic mucosal resection (P-EMR), mostly because ESD is a more complex procedure and therefore generally takes place in expert centers, generating a higher carbon footprint for patient travel 30 . An LCA reported 0.3 kg CO _2_ e for processing of GI biopsies 29 . Another study showed that use of an innovative tool called EndoFaster to analyze gastric juice during upper endoscopy instead of standard biopsy sampling reduced gastric biopsies by 50% and CO _2_ emissions by 44% 50 . Another study describing an LCA of sterile water bottles during colonoscopies concluded that emissions varied mostly per disposal method, totaling 0.2 kg per bottle for landfilling, 0.3 kg for recycling, and 0.4 kg for incineration 42 . Travel emissions ranged from 0.1–1.9 kg CO _2_ e for staff 35 37 45 to 6.6–32.0 kg CO _2_ e for patients 33 35 37 45 48 , with patient travel being a significant contributor to the carbon footprint of departments (up to 45%) or procedures such as capsule endoscopy (up to 95%) ( **Table 5s** ). Waste disposal per procedure, quantified in 11 studies 23 24 25 30 34 36 37 44 47 48 49 , ranged from 0.3–3.0 kg, with studies varying in types of waste considered (general waste, infectious waste, recyclables, sharps waste) and disposal methods used (landfill, incineration, recycling) ( Fig. 3 **, Table 6s** ).

### Analysis of carbon footprint contributions

Carbon footprint contributions varied significantly across studies. For endoscopy departments, patient and staff travel was the leading contributor, followed by single-use products and energy use. Climate control and room lighting were the primary energy sources. Waste generation played a minor role in overall emissions. For single-use products, manufacturing was the primary contributor, while for reusable products, reprocessing (decontamination) had the most impact.

### Study quality and reporting of evidence

A total of 21 (75%) studies 24 27 28 31 32 33 34 35 36 38 40 41 42 43 44 45 46 47 48 49 50 were considered to have a high risk of bias, primarily due to potential confounding factors and measurement bias caused by failure to blind study participants and/or study outcome assessors, or by omitting certain processes from the system boundary, resulting in underreporting of environmental impact ( **Table 7s** ). When applying the E-SPARE checklist criteria on reporting of endoscopy sustainability studies to all included studies, we found that 17 studies (61%) adequately reported on most (>50%) criteria ( **Table 8s** ). Study objectives, system boundaries, and emission factor sources were reported in 20 (71%), 23 (82%), and 21 (75%) studies, respectively. However, 18 studies (64%) did not provide a clear functional unit, and 19 studies (68%) provided no justification for chosen environmental impact assessment methods. Only five studies 26 31 32 35 48 (18%) reported GHG emissions according to the three emission scopes, and four studies 29 37 45 46 (14%) reported an uncertainty assessment. The quality of the LCA studies, which were additionally assessed using a pro forma quality assessment scoring system, ranged from moderate to high (66%–84%) ( **Table 9s** ). However, both internal and external validity were compromised by limited transparency. Three of ten LCA studies 29 39 42 conducted sensitivity analyses, revealing significant variability in results (up to 20%). Seven studies lacked clear justification of the functional unit, and nine studies failed to report the significance of exclusions or assumptions.

## Discussion

This systematic review highlights substantial variability in the estimated carbon emissions per GI endoscopy procedure, ranging from 5.4 to 73.2 kg CO₂e. Despite substantial differences in methodology and coverage, three consistent hotspots emerged from included studies: patient travel, energy consumption, and use of single-use products.

With approximately 134 million GI endoscopy procedures performed globally each year 51 , extrapolated annual emissions range from 727 million to 9.8 billion kg CO₂e 52 . Travel-related emissions accounted for 45%–95% of per-procedure totals, suggesting that integrating telemedicine for pre- and post-procedural consultations, where clinically appropriate, could substantially reduce this burden. In addition, variability in emissions from both patient and staff commuting highlights the potential value of decentralizing services. Locating endoscopy closer to patients’ homes, such as through satellite centers or regional hubs, may further reduce travel-related emissions while maintaining access to care. Energy use – particularly in procedure rooms and reprocessing areas – was another major contributor. One study reported a total energy consumption of 19.8 kWh per day, almost 3-fold higher than in other studies 25 . This study included energy use in pre- and post-procedure areas, while other studies excluded this from their analyses, possibly explaining this difference 26 27 28 . Transitioning to renewable energy sources, as demonstrated in selected centers, can potentially reduce energy emissions to near zero 32 . However, implementation must consider the local energy mix and institutional infrastructure. Single-use products were another major contributor. High volumes of single-use biopsy forceps, polypectomy devices, single-use endoscopes, and sterile packaging contribute significantly to material use, manufacturing emissions, and waste incineration. While single-use products have 3–10 times higher life cycle emissions than reusable products, persistent concerns around infection control and reprocessing capacity continue to drive reliance on single-use products 53 54 . Although waste generation across the included literature ranged from 0.3 to 3.0 kg per procedure, two studies that analyzed emissions at the departmental level found that waste represented less than 3% of total departmental emissions 37 48 .

Emissions varied with procedure type, use of single-use vs. reusable products, institutional waste policies, and local energy sources. Similar variability has been observed in other resource-intensive clinical environments, such as intensive care units and operating rooms 55 56 . Only 4 of the 28 studies 38 39 40 46 assessed environmental impacts beyond GHG emissions (e.g. water use, ecotoxicity, or resource depletion), and 3 studies examined the environmental footprint of entire endoscopy departments 32 37 48 . Moreover, reporting across the GHG protocol’s three emission scopes was inconsistent. Scope 3 emissions were reported in 24 studies (86%), yet coverage remained incomplete. Potentially important contributors such as pharmaceuticals and chemicals were mostly not included.

A major strength of this review is the comprehensive synthesis of the environmental impact of GI endoscopy, encompassing a broad range of environmental indicators and methodological approaches. By aligning our analysis with the GHG Protocol, we have provided a structured perspective on emissions across procedural and departmental levels. The systematic and transparent review methodology, combined with a critical appraisal of study quality, enhances the rigor and robustness of our findings.

Some limitations should also be acknowledged. Despite growing interest in the environmental sustainability of endoscopy, the current evidence base remains limited. Many studies focused narrowly on specific elements – such as waste, energy use, or individual devices – without accounting for the full procedure or departmental context. Substantial methodological heterogeneity, unclear system boundaries, and limited transparency in data sources hinder comparability. Reported footprints varied depending on data sources and regional assumptions; studies based on fossil-fuel-dominated energy mixes, including full life cycle impacts, or single-use products, generally reported higher emissions than those with narrower boundaries or cleaner energy assumptions. Differences in reprocessing protocols, waste management, and product lifespan add further uncertainty. Comparative studies often overlooked shared resource use, potentially underestimating total environmental impact. Risk of bias was assessed using the CEECAT tool, the only instrument currently targeting sustainability studies. As a 2023 prototype tool without formal validation in health care sustainability research, CEECAT raises concerns about construct validity. To address this, we operationalized the criteria for endoscopy sustainability studies, applied dual independent review with consensus, and complemented CEECAT with the ESGE E-SPARE checklist and an LCA appraisal framework to provide a broader assessment of study quality. These limitations highlight a broader methodological gap, as validated tools for assessing study quality in sustainability research are currently lacking.

Going forward, sustainable transformation of GI endoscopy must be informed by high-quality, system-wide assessments. Current research is mostly fragmented, focusing on isolated components such as waste or energy. A life cycle perspective is essential to identify trade-offs; for instance, interventions that reduce waste may inadvertently increase water or energy use.

The recent ESGE position statement on sustainability in endoscopy (E-SPARE) provides an important step toward more standardized and transparent reporting 18 . However, in our systematic review, no study reported on all E-SPARE reporting criteria. Furthermore, harmonization must extend beyond reporting alone. Standardization of assessment methods is essential to improve comparability across studies and support benchmarking of sustainability interventions across institutions and countries. Only through consistent, comprehensive measurement, can the field assess progress and identify effective decarbonization strategies. To improve the quality and comparability of future studies, environmental assessments in GI endoscopy should follow standardized methods such as LCA and the GHG Protocol, in line with the ESGE E-SPARE reporting criteria. Where feasible, studies should account for the full life cycle of products and processes, report impacts per procedure, and transparently document data sources and assumptions. Comprehensive inclusion of Scope 1, 2, and 3 emissions – particularly Scope 3 – is essential. Publishing GI-specific methodological details will further improve reproducibility and support the development of best practices.

## Conclusion

Current evidence on the environmental impact of GI endoscopy services is fragmented, methodologically inconsistent, and often limited in coverage. Emissions are dominated by patient travel, energy use, and procedure-related products, whereas waste contributes comparatively less. Broader and more standardized environmental assessments are essential to support the transition to low-carbon, sustainable GI endoscopy.

## Green Stamp Explained

This study provides a snapshot of our current understanding of the environmental impact of gastrointestinal endoscopy and the quality of the available evidence. Impressively, 28 studies have been published – most within the past 2 years – yet outcome parameters and methodological quality vary substantially. Recent ESGE guidance on reporting in this field (E-SPARE; *Endoscopy* 2025; 57: 674–688) may help reduce this heterogeneity. The findings underscore where endoscopy teams can meaningfully influence environmental impact (e.g. choice of supplies, instrument use, and waste handling) and where our influence is more limited (e.g. patient travel and energy demands). Overall, the results highlight the need for high-quality research, including practically relevant studies that compare different pathways related to endoscopy performance.

## Supplementary Material

Supplementary Material Zusatzmaterial
