# Supplementary material for: Environmental footprint of gastrointestinal endoscopy services: a systematic review
Source: Endoscopy. 2025 Dec 19;58(5):529–49. doi: 10.1055/a-2739-4080 (PMC13305580; doi:10.1055/a-2739-4080)
Supplement: Supplementary file 1 — Supplementary Material [file 10-1055-a-2739-4080_27677408.pdf]

Supplementary material

Environmental footprint of gastrointestinal endoscopy services: a systematic review

Britta Vegting, Demi Gerritsen, Ceyda B. Izci, Nicole G. M. Hunfeld, Erik M. van Raaij, Wilco van den Heuvel, Pieter J. F. de Jonge, Peter D. Siersema

Table 1s Search strategy

Database searched	Platform	Years of coverage	Search strategy
MEDLINE ALL	Ovid	1946 - Present	(Endoscopy / OR Endoscopes / OR exp Endoscopy, Digestive System / OR (endoscop* OR colonoscop* OR gastroscop* OR esophagoscop* OR esophagogastroduodenoscop* OR gastroduodenoscop* OR esophagogastroscop* OR eosophagoscop* OR duodenoscop* OR sigmoidoscop*).ab,ti,kw.) AND (* Environment OR Medical Waste Disposal / OR Climate Change/ OR Carbon Footprint/ OR Particulate Matter/ OR Radiation, Ionizing/ OR Ocean Acidification/ OR Eutrophication/ OR Fossil Fuels/ OR (((environment* OR carbon OR co2 OR co-2 OR climate*) ADJ3 (impact* OR sustain* OR footprint* OR emission* OR reduct* OR cost OR pollut*)) OR (greenhouse ADJ (effect* OR gas*)) OR waterlog* OR water-log* OR ((climat* OR global) ADJ (warming OR change OR action*)) OR (waste* ADJ3 (disposal*)) OR green*-endoscop* OR (ozone* ADJ3 (deplet* OR formation*)) OR (human ADJ3 toxicit*) OR particulate-matter* OR PM10 OR PM2-5 OR PM-10 OR PM-2-5 OR ((ionizing OR ionising) ADJ3 radiat*) OR acidificat* OR eutrophicat* OR ecotoxic* OR ecologic*-toxic* OR land-use OR land-transformation* OR (water ADJ3 (footprint* OR consumption* OR deprevat*)) OR water-use OR resource-use OR (resource* ADJ3 depletion*) OR fossil-fuel* OR soil-qualit*).ab,ti,kw. OR (sustainab* OR footprint* OR foot-print* OR environmental* OR climate* OR (green* ADJ2 endoscop*) OR ozone* OR greenhouse OR pollut*).ti.) NOT (* Indocyanine Green / OR (greenlight* OR green-light* OR indocyanine-green*).ti.) NOT (exp animals/ NOT humans/) AND english.la
Embase	Embase.c om	1971 - Present	(endoscopy/de OR endoscope/de OR 'digestive endoscope'/exp OR 'digestive tract endoscopy'/exp OR (endoscop* OR colonoscop* OR gastroscop* OR esophagoscop* OR esophagogastroduodenoscop* OR gastroduodenoscop* OR esophagogastroscop* OR eosophagoscop* OR duodenoscop* OR sigmoidoscop*):Ab,ti,kw) AND ('environmental impact'/exp OR 'environmental sustainability'/de OR 'waste disposal'/de OR 'climate change'/exp OR 'carbon footprint'/de OR 'carbon dioxide emission'/de OR 'particulate matter'/exp OR 'ionizing radiation'/de OR acidification/de OR eutrophication/de OR ecotoxicity/de OR 'land use'/de OR 'water footprint'/de OR 'resource use efficiency'/de OR 'resource depletion'/de OR 'fossil fuel'/de OR 'soil quality'/de OR (((environment* OR carbon OR co2 OR co-2 OR climate*) NEAR/3 (impact* OR sustain* OR footprint* OR emission* OR reduct* OR cost OR pollut*)) OR (greenhouse NEXT/1 (effect* OR gas*)) OR waterlog* OR water-log* OR ((climat* OR global) NEXT/1 (warming OR change OR action*)) OR (waste* NEAR/3 (disposal*)) OR green*-endoscop* OR (ozone* NEAR/3 (deplet* OR formation*)) OR (human NEAR/3 toxicit*) OR particulate-matter* OR PM10 OR PM2-5 OR PM-10 OR PM-2-5 OR ((ionizing OR ionising) NEAR/3 radiat*) OR acidificat* OR eutrophicat* OR ecotoxic* OR ecologic*-toxic* OR land-use OR land-transformation* OR (water nEAR/3 (footprint* OR consumption* OR deprevat*)) OR water-use OR resource-use OR (resource* NEAR/3 depletion*) OR fossil-fuel* OR soil-qualit*):Ab,ti,kw OR (sustainab* OR footprint* OR foot-print* OR environmental* OR climate* OR (green* NEXT/2 endoscop*) OR ozone* OR greenhouse OR pollut*):ti) NOT ('indocyanine green'/mj OR (greenlight* OR green-light* OR indocyanine-green*):ti) NOT ([animals]/lim NOT [humans]/lim) NOT ([conference abstract]/lim AND [2000-2022]/py) AND [english]/lim
Web of Science Core Collectio n	Web of Knowledg e	1975 - Present	TS=((endoscop* OR colonoscop* OR gastroscop* OR esophagoscop* OR esophagogastroduodenoscop* OR gastroduodenoscop* OR esophagogastroscop* OR eosophagoscop* OR duodenoscop* OR sigmoidoscop*)) AND (TS=(((environment* OR carbon OR co2 OR co-2 OR climate*) NEAR/2 (impact* OR sustain* OR footprint* OR emission* OR reduct* OR cost OR pollut*)) OR (greenhouse NEAR/1 (effect* OR gas*)) OR waterlog* OR water-log* OR ((climat* OR global) NEAR/1 (warming OR change OR action*)) OR (waste* NEAR/2 (disposal*)) OR green*-endoscop* OR (ozone* NEAR/2 (deplet* OR formation*)) OR (human NEAR/2 toxicit*) OR particulate-matter* OR PM10 OR PM2-5 OR PM-10 OR PM-2-5 OR ((ionizing OR ionising) NEAR/2 radiat*) OR acidificat* OR eutrophicat* OR ecotoxic* OR ecologic*-toxic* OR land-use OR land-transformation* OR (water nEAR/2 (footprint* OR consumption* OR deprevat*)) OR water-use OR resource-use OR (resource* NEAR/2 depletion*) OR fossil-fuel* OR soil-qualit*) OR TI=(sustainab* OR footprint* OR foot-print* OR environmental* OR climate* OR (green* NEAR/2 endoscop*) OR ozone* OR greenhouse OR pollut*)) NOT TI=((greenlight* OR green-light* OR indocyanine-green*)) AND DT=(article) AND LA=(english)

**Science Citation Index Expanded (1975-present) ; Social Sciences Citation Index (1975-present) ; Arts & Humanities Citation Index (1975-present) ; Conference Proceedings Citation Index- Science (1990-present) ; Conference Proceedings Citation Index- Social Science & Humanities (1990-present) ; Emerging Sources Citation Index (2005-present) No other database limits were used than those specified in the search strategies.*

Table 2s Inventory boundaries

	Pre-procedure					Procedure						Post-procedure					
**Author, (year) [ref] Country**	Patient travel	Staff travel	Bowel preparation	Vascular access	Waste	Energy use	Consumables	Capital equipment	Endoscope	Pharmaceuticals & medical gases	Waste	Food	Laundry	Histology	Energy use	Reprocessing of endoscope	Waste
**Cunha Neves et al. (2023) [22] Portugal**					X						X						X
**De Jong et al. (2023)[23] Netherlands**											X						
**Desai et al. (2024) [24] USA**					X	X					X					X	X
**Elli et al. (2024) [25] Italy**				X		X	X	X						X*	X		
**Fichtl et al. (2024) [26] Germany**						X											
**Gayam (2020) [27] USA**						X									X		
**Gordon et al. (2021) [28] USA**		X												X			
**Grau et al. (2025) [29] France**	X		X			X	X			X							
**Henniger et al. (2023) [30] Germany**					X	X	X				X				X		X
**Henniger et al. (2023) [31] Germany**							X				X						
**Jalayeri Nia et al. (2024) [32] UK**	X				X*	X*				X	X*						X*
**Jung et al. (2025) [33] South Korea**					X						X						X
**Klose et al. (2024) [34] Germany**	X	X															
**Kojima et al. (2008) [35] Japan**					X						X						X
**Lacroute et al. (2023) [36] France**	X	X		X	X	X	X	X	X	X	X	X	X		X		X
**Lämmer et al. (2025) [37] Netherlands**	X	X			X	X	X		X	X	X		X		X	X	X
**Le et al. (2022) [38] USA**						X			X							X	
**López-Muñoz et al. (2025) [39] Spain**									X							X	
**López-Muñoz et al. (2023) [40] Spain**							X										
**Lotter et al. (2025) [41] Australia**							X										
**Martin-Cabazuelo et al. (2024) [42] Spain**							X										
**Namburar et al. (2022) [43] USA**					X						X						X
**Pioche et al. (2024) [44] France**	X	X	X	X			X										X
**Pioche et al. (2024) [45] France**									X							X	
**Ribeiro et al. (2024) [46] Portugal**					X						X					X	X
**Rughwani et al. (2025) [47] India**	X			X	X	X	X	X	X	X	X		X		X	X	X
**Vaccari et al. (2018) [48] Italy**					X						X						X
**Zullo et al. (2023) [49] Italy**							X*							X*			

** = data used from previously published article*

Thieme

Table 3s Study methods

**Author, (year) [ref] Country**	**Data sources**	**Software used for impact assessment**	**Characterization method**	**Allocation method**
**Cunha Neves et al. (2023) [22] Portugal**	Data collection: on site	N/a	Unknown	N/a
**De Jong et al. (2023) [23] Netherlands**	Data collection: on site	N/a	UK Government GHG Conversion Factors for Company Reporting (2016)	N/a
**Desai et al. (2024) [24] USA**	Data collection: on site	N/a	US EPA GHGe calculator	N/a
**Elli et al. (2024) [25] Italy**	Data collection: on site, manufacturers. Secondary data source: scientific literature (Gordon et al. [28]). LCI database: Italian Higher Institute for Environmental Protection and Research (2022), emission factors report of the International Energy Agency.	Unknown	US EPA GHGe calculator	Unknown
**Fichtl et al. (2024) [26] Germany**	Data collection: on site.	N/a	German electricity generation average	N/a
**Gayam (2020) [27] USA**	Data collection: on site	N/a	Unknown	N/a
**Gordon et al. (2021) [28] USA**	Data collection: on site. LCI database: EcoInvent database, chemical life cycle collaborative	SimaPro software v8.5.2.3	TRACI (EPA), CLiCC LCIA Estimate tool	APOS (attributional)
**Grau et al. (2025) [29] France**	Data collection: on site, material composition analysis. Secondary data source: scientific literature. LCI database: EcoInvent v3.8, emission factors reported by ADEME	Granta Design	ADEME	Unknown
**Henniger et al. (2023) [30] Germany**	Data collection: on site. LCI database: EcoInvent 3.8	Unknown	UK Government GHG Conversion Factors for Company Reporting (2022, v2.0).	Unknown
**Henniger et al. (2023) [31] Germany**	Data collection: on site. LCI database: EcoInvent v3.8	Unknown	UK Government GHG Conversion Factors for Company Reporting.	Unknown
**Jalayeri Nia et al. (2024) [32] UK**	Data collection: on site.	Unknown	Conversion factors from the Department for Energy Security and Net Zero (UK)	Unknown
**Jung et al. (2025) [33] South Korea**	Data collection: on site, manufacturers. Secondary data source: scientific literature (Gordon et al[27]). LCI database: Italian Higher Institute for Environmental Protection and Research (2022)	N/a	N/a	N/a
**Klose et al. (2024) [34] Germany**	Data collection: on site	N/a	GHG protocol conversion factors (2023)	N/a
**Kojima et al. (2008) [35] Japan**	Data collection: on site	N/a	N/a	N/a
**Lacroute et al. (2023) [36] France**	Data collection: on site. Secondary data source: monetary ratios. LCI database: EcoInvent, AGRIBALYSE	Bilan Carbon tool v8.7.1	ADEME Carbon base, Guide sectorial BEGES Sante	Unknown
**Lämmer et al. (2025) [37] Netherlands**	Data collection: on site. Secondary data source: manufacturers. LCI database: EcoInvent 3.9	SimaPro software v9	ReCiPe 2016	Unknown
**Le et al. (2022) [38] USA**	Data collection: on site. Secondary data source: manufacturers, cystoscope (Davis et al). LCI database: EcoInvent 3.8	SimaPro software v9.1.1, Epi Suite 4.11.	ReCiPe 2016, USEtox 2.12	Unknown

**López-Muñoz et al. (2025) [39] Spain**	Data collection: on site, MCA. Secondary data source: EcoInvent, Agribalyse, EF secondary data. LCI database: UK Government GHG Conversion Factors for Company Reporting.	OpenLCA v2.0.3	EF v3.0	Unknown
**López-Muñoz et al. (2023) [40] Spain**	Data collection: on site, MCA. Secondary data source: scientific literature. LCI database: EcoInvent v3.8.1	OpenLCA v1.11	EF v3.0	Attributional analysis
**Lotter et al. (2025) [41] Australia**	Data collection: on site	Unknown	Unknown	N/a
**Martin-Cabazuelo et al. (2024) [42] Spain**	Data collection: on site, MCA. Secondary data source: scientific literature, assumption. LCI database: EcoInvent v3.8.1.	OpenLCA v1.11	EF v3.0	Unknown
**Namburar et al. (2022) [43] USA**	Data collection: on site.	N/a	N/a	N/a
**Pioche et al. (2024) [44] France**	Data collection: on site, MCA, manufacturers. Secondary data source: scientific literature. LCI database: CES EduPack 2022	Ansys Granta Edupack software	ADEME	Unknown
**Pioche et al. (2024) [45] France**	Data collection: on site, MCA. Secondary data source: scientific literature, monetary ratio, assumption. LCI database: EcoInvent v3.8.1, ADEME	SimaPro software v9.3	CML-IA baseline v3.07	Unknown
**Ribeiro et al. (2024) [46] Portugal**	Data collection: on site.	N/a	N/a	N/a
**Rughwani et al. (2025) [47] India**	Data collection: on site, manufacturers. LCI database: separate Emission Factors used	Excel, Microsoft	N/a	Unknown
**Vaccari et al. (2018) [48] Italy**	Data collection: Italian Hospital	N/a	N/a	N/a
**Zullo et al. (2023) [49] Italy**	Data collection: on site. Secondary data source: biopsy processing data from Gordon et al. [27]	Unknown	Institute for Sustainability Leadership of the University of Cambridge, IPCC	Unknown

ADEME, agence de la transition écologique; APOS, allocation at the point of substitution; BEGES, bilan d'émissions de gaz à effet de serre; CLiCC, chemical life cycle collaborative; CML-IA, institute for environmental sciences impact assessment; EF, environmental footprint; EPA, environmental protection agency; GHG, greenhouse gas; IPCC, Intergovernmental Panel on Climate Change; LCI, life cycle inventory; LCIA, life cycle impact assessment; MCA, material composition analysis; n/a, not applicable; TRACI, tool for the reduction and assessment of chemical and other environmental impacts; USA, United States of America; UK, United Kingdom

Table 4s Energy use in the endoscopy department

Study	Desai et al. (2024) [24]	Elli et al. (2024) [25]	Fichtl et al. (2024) [26]	Gayam et al. (2020) [27]	Rughwani et al. (2025) [47]
**Country**	USA	Italy	Germany	USA	India
**Endoscopy machine [kWh]**		0.7	0.2-0.4	0.7*	
**Monitors & computers [kWh]**		0.2			
**Anesthesia machine [kWh]**		0.3		0.3*	
**Room lighting [kWh]**		1.2		1.2*	
**Climate control [kWh]**		2.5			
**Reprocessing wash machines [kWh]**		0.6		0.6*	
**Entire department [kWh/day]**	**277.1**			**111.6**	
**Overall energy consumption per procedure [kWh]**	**19.8****	**5.5**	**0.2-0.4**	**2.8***	**4.0**

kWh, kilowatt Hour; USA, United States of America

** Data not directly provided by article, calculations based on their provided average of 40 endoscopies per day.*

*** Data not directly provided by article, calculation based on their provided average for 100 procedures.*

Table 5s Patient and staff travel emissions in gastrointestinal endoscopy

Study	Jalayeri Nia et al. (2024) [32]	Klose et al. (2024) [34]	Lacroute et al. (2023) [36]	Pioche et al. (2024) [44]	Rughwani et al. (2025) [47]
**Country**	UK	Germany	France	France	India
**Scope**	CCE	Outpatient procedures	Outpatient procedures	SBCE	Outpatient procedures
**Patient travel CO_2e/procedure [kg]**	6.6-17.1	10.7	15.4	18.4	32
**Staff travel CO_2e /procedure [kg]**	n/a	0.8	1.9	0.1	n/a

CCE, colon capsule endoscopy; CO_2e, carbon dioxide equivalent; n/a, not assessed; kg, kilogram, SBCE, small-bowel capsule endoscopy; UK, United Kingdom

Table 6s Waste generation in the endoscopy department

Mean waste and waste components							
**Author (year) [ref.]**	**Country**	**Amount of procedures**	**Infectious waste (%)**	**Sharps waste (%)**	**General waste (%)**	**Recyclables (%)**	**Waste, mean (kg/procedure)**
**Kojima et al. (2008) [35]**	**Japan**	307	68.9-92.9	0-7.1	0-19.0	0-4.9	**0.3**
**Vaccari et al. (2018) [48]**	**Italy**	Unknown					**0.5**<br>**3.1/bed**
**Namburar et al. (2022) [43]**	**USA**	278	28		64	9	**2.3**
**Cunha Neves et al. (2023) [22]**	**Portugal**	535	41.2-61.2		38.8-50.9	0-7.2	**0.5-1.0**
**De Jong et al. (2023) [23]**	**Netherlands**	36			85-91.1	8.9-9.6	**0.9-1.0**
**Lacroute et al. (2023) [36]**	**France**	8,524					**1.5**
**Ribeiro et al. (2024) [46]**	**Portugal**	241	74.1	0.9	7.2	17.8	**1.8**
**Grau et al. (2025) [29]**	**France**	359					**1.7-2.3**
**Desai et al. (2024) [24]**	**USA**	450	24	4	57.6	14.4	**3.0**
**Rughwani et al. (2025) [47]**	**India**	3,873	64.8	1.1	21.8	12.2	**0.5**
**Jung et al. (2025) [33]**	**South Korea**	3,922					**1.3**

Categorized using the World Health Organization (WHO) standard healthcare waste categories, excluding pathological, chemical and pharmaceutical waste, as no study examined these categories. Kg, kilogram; ref., reference; USA, United States of America.

Table 7s Risk of bias assessment for included studies using Collaboration for Environmental Evidence Critical Appraisal Tool (CEECAT)

Author (year) [ref] Country	Criterion 1: Risk of Confounding biases	Criterion 2: Risk of post-intervention /exposure selection biases	Criterion 3: Risk of misclassified comparison biases	Criterion 4: Risk of performance biases	Criterion 5: Risk of detection biases	Criterion 6: Risk of outcome reporting biases	Criterion 7: Risk of outcome assessment biases	Overall judgement
	Operationalization by team							
	Specific factors (e.g. procedure type, device reuse, energy mix) affecting results	Differences in included procedures or settings after intervention/audit	Used for non-interventional studies. Incorrect or inconsistent classification of comparators	Used for interventional studies. Variations in staff behavior or protocols influencing outcomes	Inconsistent or non-standardized outcome measurement	Selective or incomplete reporting of environmental outcomes	Outcome assessors influenced by knowledge of exposure/intervention	Combined risk-of-bias rating based on criteria 1-7
Cunha Neves et al. (2023) [22] Portugal	Low risk of bias	Low risk of bias	Not Applicable	Low risk of bias	Low risk of bias	Low risk of bias	Medium risk of bias	Medium risk of bias
De Jong et al. (2023) [23] Netherlands	High risk of bias	Low risk of bias	Not Applicable	Low risk of bias	Medium risk of bias	Low risk of bias	High risk of bias	High risk of bias
Desai et al. (2024) [24] USA	Low risk of bias	Low risk of bias	Medium risk of bias	Not Applicable	Medium risk of bias	Low risk of bias	Medium risk of bias	Medium risk of bias
Elli et al. (2024) [25] Italy	High risk of bias	Medium risk of bias	Medium risk of bias	Not Applicable	Low risk of bias	High risk of bias	Medium risk of bias	Medium risk of bias
Fichtl et al. (2024) [26] Germany	High risk of bias	Low risk of bias	Not Applicable	Low risk of bias	Medium risk of bias	Low risk of bias	High risk of bias	High risk of bias
Gayam (2020) [27] USA	High risk of bias	Medium risk of bias	High risk of bias	Not Applicable	Medium risk of bias	High risk of bias	High risk of bias	High risk of bias
Gordon et al. (2021) [28] USA	Low risk of bias	Medium risk of bias	Not Applicable	Medium risk of bias	Medium risk of bias	Medium risk of bias	Medium risk of bias	Medium risk of bias
Grau et al. (2025) [29] France	Low risk of bias	Low risk of bias	Not Applicable	Low risk of bias	Low risk of bias	medium risk of bias	Medium risk of bias	medium risk of bias
Henniger et al. (2023) [30] Germany	Low risk of bias	Medium risk of bias	Not Applicable	Medium risk of bias	Medium risk of bias	Medium risk of bias	Medium risk of bias	High risk of bias
Henniger et al. (2023) [31] Germany	High risk of bias	Medium risk of bias	Not Applicable	Low risk of bias	Medium risk of bias	Low risk of bias	Medium risk of bias	High risk of bias
Jalayeri Nia et al. (2024) [32] UK	High risk of bias	Medium risk of bias	Medium risk of bias	Not Applicable	Medium risk of bias	Medium risk of bias	Medium risk of bias	High risk of bias
Jung et al. (2025) [33] South Korea	high risk of bias	Low risk of bias	Medium risk of bias	Not Applicable	Medium risk of bias	Low risk of bias	Medium risk of bias	high risk of bias
Klose et al. (2024) [34] Germany	High risk of bias	Medium risk of bias	Medium risk of bias	Not Applicable	Medium risk of bias	Medium risk of bias	Medium risk of bias	High risk of bias
Kojima et al. (2008) [35] Japan	High risk of bias	Low risk of bias	Not Applicable	High risk of bias	Medium risk of bias	Medium risk of bias	Medium risk of bias	High risk of bias
Lacroute et al. (2023) [36] France	Low risk of bias	Low risk of bias	Low risk of bias	Not Applicable	Medium risk of bias	Low risk of bias	Low risk of bias	Medium risk of bias
Lämmer et al. (2025) [37] Netherlands	high risk of bias	Low risk of bias	Low risk of bias	Not Applicable	Medium risk of bias	Low risk of bias	Medium risk of bias	high risk of bias

**Le et al. (2022) [38] USA**	Low risk of bias	Low risk of bias	Medium risk of bias	Not Applicable	Low risk of bias	Low risk of bias	Medium risk of bias	Medium risk of bias
**López-Muñoz et al. (2025) [39] Spain**	High risk of bias	Low risk of bias	Medium risk of bias	Not Applicable	Medium risk of bias	Medium risk of bias	Medium risk of bias	High risk of bias
**López-Muñoz et al. (2023) [40] Spain**	High risk of bias	Low risk of bias	Not Applicable	Low risk of bias	Low risk of bias	Medium risk of bias	Medium risk of bias	High risk of bias
**Lotter et al. (2025) [41] Australia**	high risk of bias	Low risk of bias	high risk of bias	Not Applicable	Low risk of bias	Low risk of bias	Medium risk of bias	high risk of bias
**Martín-Cabazuelo et al. (2024) [42] Spain**	High risk of bias	Low risk of bias	Medium risk of bias	Not Applicable	Medium risk of bias	Medium risk of bias	Medium risk of bias	High risk of bias
**Namburar et al. (2022) [43] USA**	High risk of bias	Medium risk of bias	Medium risk of bias	Not Applicable	Medium risk of bias	Low risk of bias	Medium risk of bias	High risk of bias
**Pioche et al. (2024) [44] France**	High risk of bias	Low risk of bias	Medium risk of bias	Not Applicable	Medium risk of bias	Medium risk of bias	Medium risk of bias	High risk of bias
**Pioche et al. (2024) [45] France**	High risk of bias	Low risk of bias	Low risk of bias	Not Applicable	Medium risk of bias	Medium risk of bias	Medium risk of bias	High risk of bias
**Ribeiro et al. (2024) [46] Portugal**	High risk of bias	Low risk of bias	Medium risk of bias	Not Applicable	Medium risk of bias	Medium risk of bias	Medium risk of bias	High risk of bias
**Rughwani et al. (2025) [47] India**	high risk of bias	Low risk of bias	Low risk of bias	Not Applicable	Medium risk of bias	Low risk of bias	Medium risk of bias	high risk of bias
**Vaccari et al. (2018) [48] Italy**	High risk of bias	Low risk of bias	Low risk of bias	Not Applicable	Low risk of bias	Medium risk of bias	Medium risk of bias	High risk of bias
**Zullo et al. (2023) [49] Italy**	High risk of bias	Low risk of bias	Medium risk of bias	Not Applicable	Medium risk of bias	Medium risk of bias	Medium risk of bias	High risk of bias

Ref, reference; UK, United Kingdom; USA, United States of America.

Each CEECAT domain was adapted to GI endoscopy sustainability studies: study design (LCA, waste audit, carbon footprinting) was assessed for methodological appropriateness; scope and system boundaries for inclusion of relevant stages; data quality for completeness and reliability; analysis transparency for clarity of methods and assumptions; uncertainty/sensitivity for variability in energy use, product lifespan, or waste handling; conflicts of interest for potential stakeholder influence; and reporting completeness for absolute and normalized environmental impacts. Scoring followed these criteria to ensure consistent, transparent risk-of-bias assessment.

Table 8s Quality assessment for included studies using ESGE E-SPARE checklist

		[22]	[23]	[24]	[25]	[26]	[27]	[28]	[29]	[30]	[31]	[32]	[33]	[34]	[35]	[36]	[37]	[38]	[39]	[40]	[41]	[42]	[43]	[44]	[45]	[46]	[47]	[48]	[49]
**Introduction**	A study hypothesis or objective is stated																												
**Methods**	The functional unit is defined																												
	The study (system) boundary is clearly defined																												
	The clinical setting, care pathway or departmental characteristics under analysis are clearly described																												
	The methodological approach used to assess environmental impacts is explicitly stated and justified (e.g. carbon footprinting, LCA)																												
	The environmental impacts chosen for assessment are defined and justified, using standard terminology and units of measurement																												
	Assumptions or exclusions are clearly stated and justified																												
	An inventory of all processes withing the system boundary is compiled and available to review																												
	Allocation methods are described and justified																												
	Emission factors sources are stated																												
**Results**	Endoscopic procedures or devices included in the analysis are characterized																												
	GHG emissions are reported according to the three healthcare Scopes (1, 2 and 3)																												

	An uncertainty assessment is conducted																												

LEGEND	
	Fully assessed
	Partially assessed
	Not assessed
	Not applicable

ESGE, European Society of Gastroenterology; E-SPARE, endoscopic sustainability primary reporting essentials; GHG, greenhouse gas; LCA, life cycle assessment.

Table 9s Quality assessment of included studies describing LCAs

Appraisal criteria	Indicator(s)	Operationalization by our research team - adapted from Kouwenberg et al. (2024) [20]	Gordon (2021) [28]	Grau (2025) [29]	Lämmer (2025) [37]	Le (2022) [38]	López Muñoz (2025) [39]	López Muñoz (2023) [40]	Lotter (2025) [41]	Martín Cabazuelo (2024) [42]	Pioche (2024) [44]	Pioche (2024) [45]
Phase 1: goal and scope (13 points)												
Study goal is clearly stated, including the study's rationale **(1)** intended application **(1)** and intended audience **(1)**	Transparency		2	2	2	2	2	3	2	2	2	2
LCA method is clearly stated **(1)**	Transparency	If the term LCA was not explicitly used, this item scored zero points	1	1	1	1	1	1	1	1	1	1
Functional unit is clearly defined and measurable **(1)** justified **(1)** and consistent with the study's intended application **(1)**	Consistency	No points were subtracted if the term “functional unit” was not explicitly used. Points were given based on a clear description of the unit of analysis. In case no intended application was mentioned (scoring item 1), consistency with the study's aim was assessed.	3	2	3	2	2	1	2	3	2	2
The system studied is adequately described with clearly stated system boundaries **(1)**, life cycle stages **(1)**, and appropriate justification of any omitted stages **(1)**	Transparency; bias	Points for appropriate justification of any omitted stages were not given if the study listed only excluded elements, without an explanation of why these were excluded.	3	2	2	2	3	3	3	3	3	3
The system covers production **(1)** use/reuse **(1)** and disposal **(1)** of materials and energy	Internal validity, completeness	The original assessment tool included: “half mark if only for energy and vice versa,” which was unclear for our reviewers and left out of the assessment.	2	2	2	1,5	1,5	2	1	1,5	1,5	1,5
Phase 2: Inventory analysis (7 points)												
The data collection process is clearly explained, including the source(s) of foreground material weights and energy values **(1)**, the source(s) of reference data (e.g. inventory database) **(1)** and what data are included (e.g. production and disposal of unit processes **(1)**	Transparency; internal validity		3	2	3	3	3	3	3	3	3	2

Representativeness of the data is discussed **(1)**, differences in electricity generating mix are accounted for **(1)** and the potential significance of exclusions or assumptions is addressed **(1)**	Internal validity; external validity	Point for electricity generating mix given if analyses were adjusted for local energy mix or if sensitivity to different energy mixes was assessed. Point for representativeness given only when explicitly mentioned with regard to either geographic, temporal, or technological representativeness, e.g., when prices were deflated. If geographical representativeness was only addressed in the context of the energy mix, only one point was given for "differences in electricity generating mix are accounted for." Point for addressing the potential significance of exclusions or assumptions given only if potential significance was explicitly stated, i.e., whether it potentially led to an under- or overestimation.	3	2	0	1	0	1	1	2	1	1
allocation procedures, where necessary, are described and appropriately justified **(1)**: mark given if no allocation was used	Transparency; bias	This item was given a score of 1 if no substantial allocation was deemed necessary in the study.	1	1	1	1	1	1	1	1	1	1
Phase 3: Impact assessment (6 points)												
impact categories **(1)**, characterization method **(1)**, and software used **(1)** are documented transparently	Transparency	Given that all articles mentioned the term "carbon footprint" (because this was included in the literature search), 1 point was always given for "impact categories."	3	2	3	3	3	3	0	3	3	3
Results are clearly reported in the context of the functional unit **(1)** (0.5 if graphically, 0 if only normalized results were reported)	Consistency; transparency	If no functional unit was described in phase 1, this item was judged based on the way results were presented in general.	1	1	1	1	1	1	1	1	1	1
A contribution analysis is performed and clearly reported **(1)** and hotspots are identified **(1)**		A point was given for contribution analysis if the results were summed up and presented as a total footprint.	2	2	2	2	2	1	2	2	2	2
Phase 4: Interpretation (9 points)												
Conclusions are consistent with the goal and scope **(1)** and the potential impact of omissions or assumptions on the study's outcomes are described **(1)**	Internal validity; consistency	If no goal and scope were described earlier, this item was judged based on the clearness of the provided conclusion(s) in general.	1	1	1	2	2	2	1	2	2	1

Results are contextualized through the use of sensitivity analysis **(1)** and uncertainty analysis **(1)**	Internal validity	If the study did not explicitly mention “sensitivity” or “uncertainty analysis,” but presented ranges or standard deviations: 1 point was given for “uncertainty analysis.”	1	0	0	1	0	0	0	0	0*	1
Limitations are adequately discussed **(1)** and the potential impact of omissions or assumptions on the study's outcomes are described **(1)**	Bias	Only points given when the potential impact of the omission on the study’s outcomes were explicitly mentioned, i.e., whether the omission likely led to an under- or overestimation.	1	1	1	2	2	1	2	2	2	1
The assessment has been critically appraised (peer review if journal article or independent, external critical review if report/thesis(**(1)**	Bias	No point was given in the case of a letter to the editor or a commentary because these are generally not peer-reviewed. However, if an included letter is peer-reviewed, 1 point will be given.	1	1	1	1	1	1	1	1	1	1
Source(s) of funding and any potential conflict(s) of interest are disclosed **(1)** and are unlikely to be a source of bias **(1)**	Bias	No point was given for the first item if only conflict(s) of interest were disclosed but no source(s) of funding were reported.	0	2	2	2	2	2	2	2	0	1
		**TOTAL (out of 35)**	**28**	**24**	**25**	**27,5**	**26,5**	**26**	**23**	**29,5**	**25,5**	**24,5**
		Percentage	80%	69%	71%	79%	76%	74%	66%	84%	73%	70%

Based on: Drew, J. et al. (1997)[18] and Weidema, B. P. et al. (1997)[19]. LCA, life cycle assessment; SBCE, small bowel capsule endoscopy

** The article mentioned that a sensitivity analysis was performed, but no data is shown in the article.*

Fig. 1s PRISMA flow diagram of studies included and excluded in the systematic review

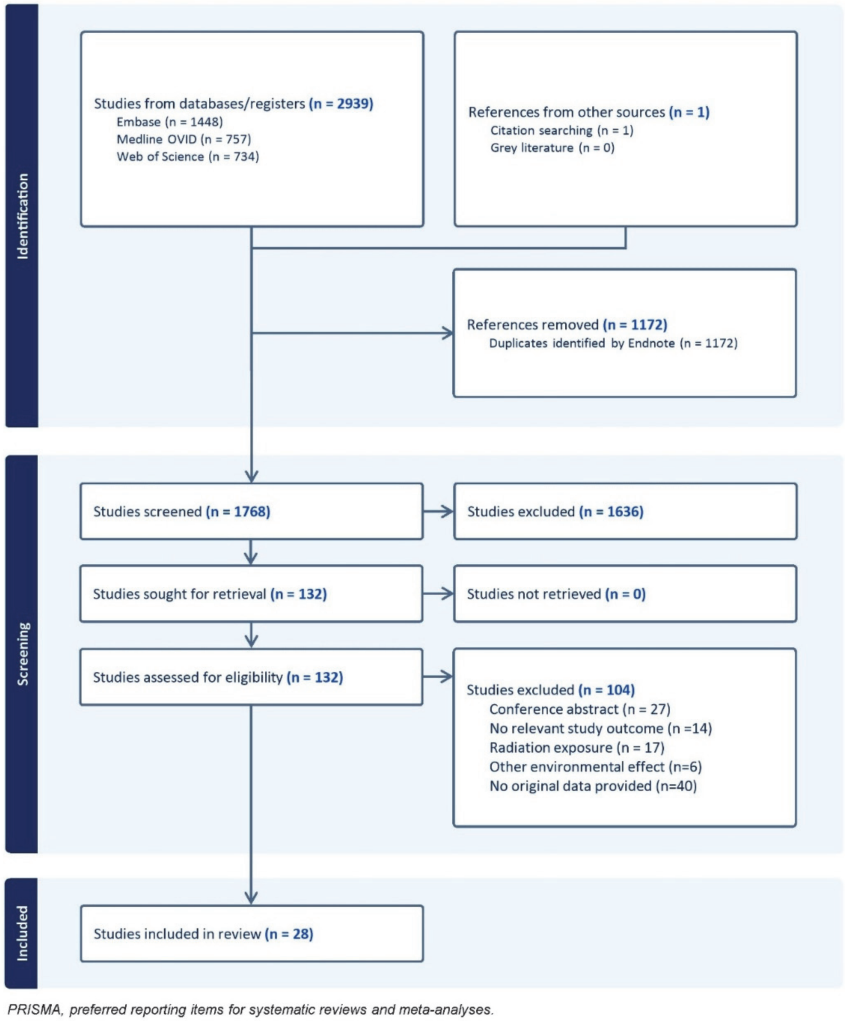

PRISMA, preferred reporting items for systematic reviews and meta-analyses.
